# Membrane Tension Inhibits Lipid Mixing by Increasing the Hemifusion Stalk Energy

**DOI:** 10.1021/acsnano.3c04293

**Published:** 2023-09-05

**Authors:** Petr Shendrik, Gonen Golani, Raviv Dharan, Ulrich S. Schwarz, Raya Sorkin

**Affiliations:** † School of Chemistry, Raymond & Beverly Sackler Faculty of Exact Sciences, Tel Aviv University, Tel Aviv, 6997801, Israel; ‡ Institute for Theoretical Physics and BioQuant Center for Quantitative Biology, Heidelberg University, 69120, Heidelberg, Germany; § Center of Physics and Chemistry of Living Systems, Tel Aviv University, Tel Aviv, 6997801, Israel

**Keywords:** membrane fusion, tension, micropipette aspiration, optical tweezers, continuum elasticity

## Abstract

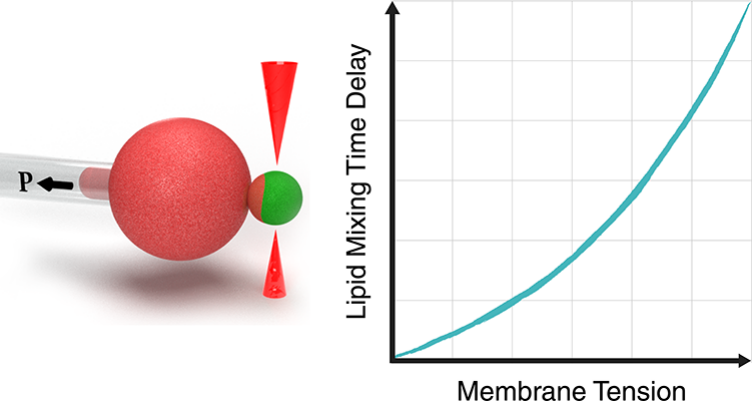

Fusion of biological membranes is fundamental in various physiological events. The fusion process involves several intermediate stages with energy barriers that are tightly dependent on the mechanical and physical properties of the system, one of which is membrane tension. As previously established, the late stages of fusion, including hemifusion diaphragm and pore expansions, are favored by membrane tension. However, a current understanding of how the energy barrier of earlier fusion stages is affected by membrane tension is lacking. Here, we apply a newly developed experimental approach combining micropipette-aspirated giant unilamellar vesicles and optically trapped membrane-coated beads, revealing that membrane tension inhibits lipid mixing. We show that lipid mixing is 6 times slower under a tension of 0.12 mN/m compared with tension-free membranes. Furthermore, using continuum elastic theory, we calculate the dependence of the hemifusion stalk formation energy on membrane tension and intermembrane distance and find the increase in the corresponding energy barrier to be 1.6 *k*_B_*T* in our setting, which can explain the increase in lipid mixing time delay. Finally, we show that tension can be a significant factor in the stalk energy if the pre-fusion intermembrane distance is on the order of several nanometers, while for membranes that are tightly docked, tension has a negligible effect.

Membrane fusion is fundamental in numerous developmental, physiological, and pathological processes, including fertilization, muscle formation, enveloped virus infection, and neural activity.^1^ While these processes differ in their time and length scales, the physical transformations lipid bilayers undergo during the fusion process are similar and involve a series of well-established intermediate steps:^2−5^ membrane contact, hemifusion stalk (“stalk” hereafter) formation, and fusion pore expansion. These steps can be experimentally detected as two subsequent mixing events: lipid mixing following stalk formation and content mixing following the fusion pore expansion.^6^ The free energy accumulated in the intermediate states defines the fusion energy barriers and determines the rate of the two mixing steps.

The mechanical and physical properties of the fusing membranes and their surroundings affect the energy of the fusion intermediates. In some cases, the same property may have opposite effects at different stages, increasing the energy of one and decreasing the energy of the other. A prominent example of such a conflict is the membrane lipid composition, namely, the lipid’s intrinsic curvature. Lipids with positive intrinsic curvature, such as lysophosphatidylcholine (LPC), inhibit lipid mixing^7^ but accelerate content mixing.^8^ Another conflicting factor is membrane tension (“tension” for brevity); however, unlike the well-documented role of lipid composition,^7,9−13^ the effect of tension is much less understood.

Tension is the free energy per unit area needed to stretch a flat membrane element, either by smoothing membrane undulations, pulling an area from a reservoir, or lipid stretching/compressing in the lateral direction.^14,15^ Tension in cellular membranes can originate from osmotic pressure difference, membrane–cytoskeleton interaction, and substrate adhesion.^16^ Tension varies across orders of magnitude within an individual cell, ranging from 0.005–0.015 mN/m in the inner membrane compartments, such as the endoplasmic reticulum and Golgi,^17^ to 0.1 mN/m in the plasma membrane.^18^ It also varies between cell types; for example, membrane tension can be as high as 0.45 mN/m in migrating cells^19,20^ due to membrane flow and friction with the substrate and cytoskeleton anchors. The maximal tension a typical lipid membrane can sustain before rupture is approximately 3–4 mN/m.^21^

The fusion of tense membranes is energetically favorable overall, as it relaxes membrane stretching stress. However, similarly to lipid intrinsic curvature, tension also has a dual effect on the fusion energy barriers. Diaphragm expansion and fusion pore formation are favored at high tension since both involve release of lipids from the fusion site to the surrounding reservoir, relaxing the stress.^16^ This effect was computationally studied^22−24^ and observed experimentally.^25−27^ In contrast, the initial steps leading to the merger of the proximal monolayers and stalk formation are inhibited by tension because stalk formation involves pulling additional lipids from the surrounding membranes, which is energetically unfavorable.^16^ In line with this is the observation of high tension blocking the lipid mixing step in hemagglutinin-mediated membrane fusion.^28^ However, despite the large variability of membrane tension in cellular membranes and the importance of membrane fusion, the effect of tension on the lipid mixing step has not been theoretically or experimentally systematically addressed before.

Here we combine optical tweezers, micropipette aspiration, and confocal fluorescence microscopy to manipulate and monitor the hemifusion processes of membranes under tension. We experimentally demonstrate that the lipid mixing time delay increases with tension and that the lipid mixing energy barrier agrees with the theoretically predicted change in the stalk formation energy. We further predict that the mean distance between the membranes in the pre-fusion configuration determines the relative contribution of tension to the stalk energy, with larger separation resulting in a larger contribution.

## Results and Discussion

### A New Method for Testing the Effect of Tension on Lipid Mixing

**Figure 1:**
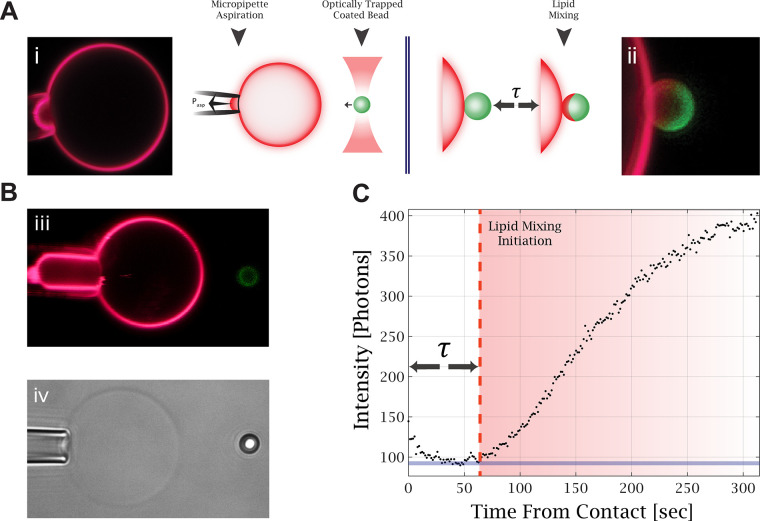
Experimental setup for lipid mixing measurements. (A) Illustration of the experiment. The optically trapped membrane-coated bead is brought into contact with the aspirated GUV (i), and confocal fluorescence microscopy scans are acquired continuously to monitor the fluorescence change caused by lipid mixing (ii). (B) (iii) Confocal fluorescence image of an aspirated GUV under high aspiration, as can be seen from the large aspirated “tongue” compared to image (i). (iv) Bright-field image of an aspirated GUV and an optically trapped bead. (C) Fluorescence intensity profile on the membrane-coated bead contact edge with the GUV. The time delay to lipid mixing is measured from the contact time to the time the fluorescence intensity increases on the bead.

We introduced a new setup that combines micropipette aspiration, optically trapped membrane-coated beads, and confocal fluorescence imaging. An aspirated giant unilamellar vesicle (GUV) and an optically trapped membrane-coated bead are brought into contact to initiate the hemifusion process, as illustrated in Figure 1A. This approach allows us to control the tension of the aspirated GUV and the membrane composition of both membranes. In addition, we precisely controlled and measured the force between the GUV and the bead. Figure 1B demonstrates tension manipulation in the GUV, manifested by changes in the length of the aspirated membrane in the micropipette. Figure 1C shows the fluorescence intensity profile over time at the bead contact point with the GUV. From such measurements, we find the lipid mixing time delay, τ, which is the time from the membrane contact to the initiation of lipid mixing. This time is measured from the contact time to the time fluorescence intensity increases on the bead.

### Method Validation

To validate our method, we performed control experiments with lipids of different intrinsic curvatures, which have a well-documented effect on the lipid mixing time delay. The early fusion stages are characterized by a strong negative splaying of the lipid’s tails. Therefore, membranes with a higher fraction of lipids with positive intrinsic curvature, such as LPC, inhibit stalk formation, while lipids with negative intrinsic curvature, such as oleic acid and cholesterol, promote it.^7,12,29,30^

**Figure 2:**
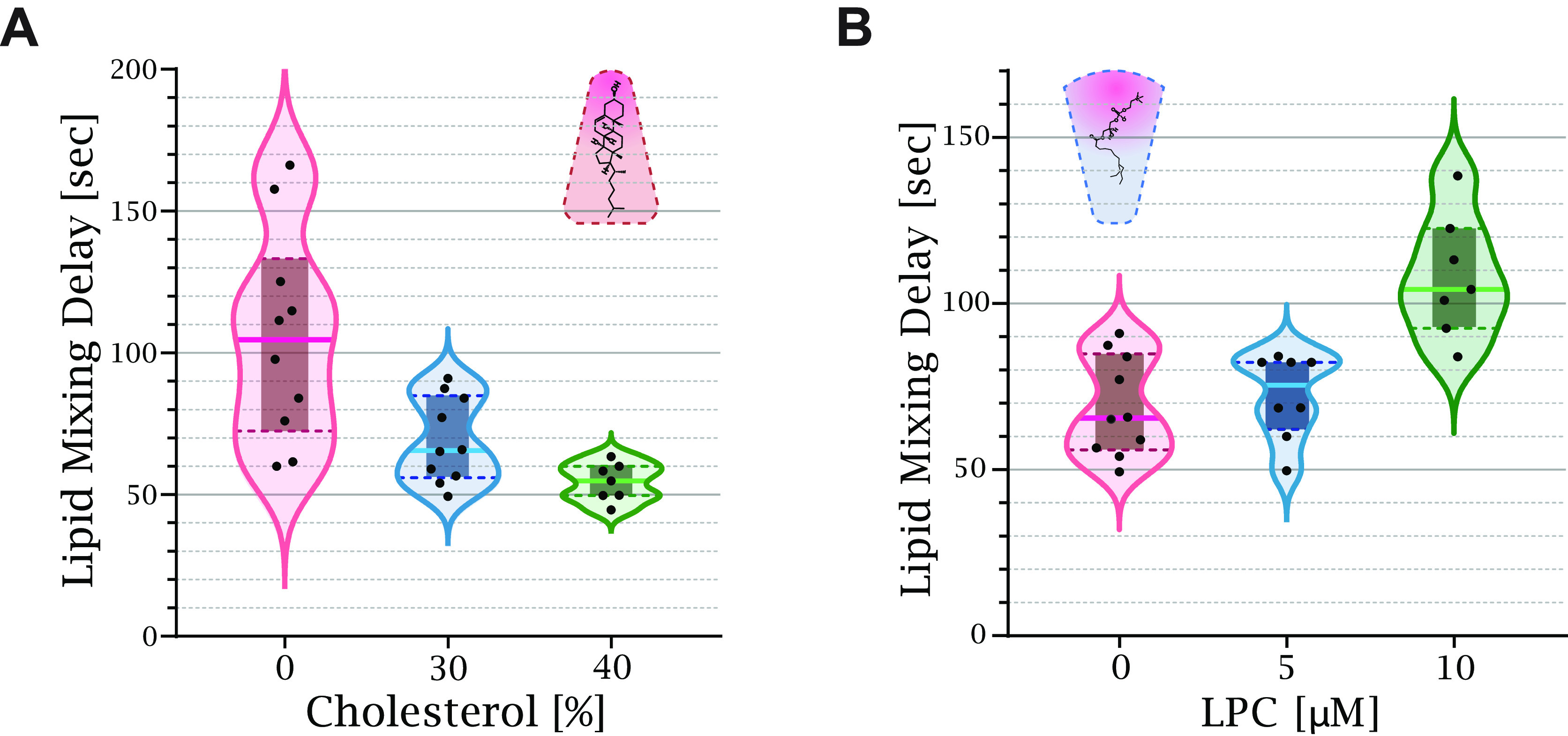
Lipid intrinsic curvature affects the lipid mixing time delay. (A) Increasing the ratio between cholesterol and DOPC in the membrane reduced the time to lipid mixing. The solid line of each box plot is the mean lipid mixing time delay. The lipid mixing time delay of 0% cholesterol is 105 ± 28 s [*n* = 10], 30% is 69 ± 11 s [*n* = 10], and 40% is 54 ± 6 s [*n* = 7]. (B) The external addition of LPC increased the lipid mixing time delay from 69 ± 11 s [*n* = 10] at 0 μmol of LPC to 75 ± 9 s [*n* = 8] at 5 μmol and 108 ± 18 s [*n* = 7] at 10 μmol.

Figure 2 depicts the effect of lipid intrinsic curvature on the lipid mixing time delay. Increasing the cholesterol concentration from 0% to 40% decreased the lipid mixing time delay from 105 ± 28 s to 54 ± 6 s (Figure 2A). On the other hand, increasing the LPC concentration in the buffer from 0 to 10 μM increased the lipid mixing time delay from 69 ± 11 s to 108 ± 17 s for membranes containing 30% cholesterol (Figure 2B). Measurements with an LPC concentration higher than 20 μM resulted in no lipid mixing during the experiment (10 min). To conclude, our control experiments agree well with numerous previous studies, demonstrating the validity of our new experimental approach.

### Tension Inhibits Lipid Mixing between a GUV and a Membrane-Coated Bead

**Figure 3:**
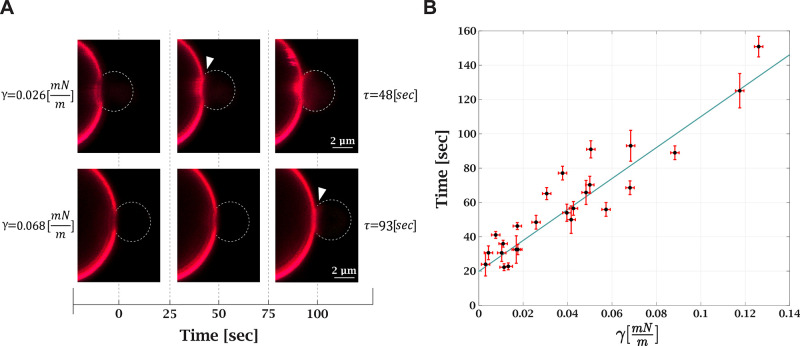
Membrane tension increases lipid mixing time delay. (A) Confocal fluorescence microscopy images of lipid mixing under different tensions. The same GUV was used in both measurements. The first frame is the contact between the bead and GUV; the white arrow points to the frame with the lipid mixing initiation. Higher tension (bottom images) increases the lipid mixing time delay. (B) Lipid mixing time delay as a function of tension (25 measurements, 12 GUVs in 8 independent experiments; for 10 GUVs, multiple measurements were performed). Black dots are the experimental results, and the solid blue line is a linear trend with a 903 ± 142 m·s/mN slope.

Following the validation of our method, we measured the effect of tension on the lipid mixing time delay. We compared the lipid mixing time delay under different tensions, ranging from 0.003 ± 0.002 to 0.125 ± 0.002 mN/m. Under a high tension of 0.3 mN/m, no lipid mixing events were observed during the experiment (10 min). The measurements were also performed on loose vesicles and at high Ca^2+^ concentration, resulting in a very short lipid mixing time delay (see SI, Movie A, at 20 mM Ca^2+^). Figure 3A depicts the effect of tension on the lipid mixing time delay. Tension increase resulted in a longer lipid mixing time delay for the same aspirated GUV (see SI, Movie B). The relation between lipid mixing time delay and tension is shown in Figure 3B. At the lowest measured tension, the lipid mixing time is 23 ± 5 s, and at the highest, 150 ± 6 s. To conclude, we found that tension variation in the physiological range increases the time from membrane contact to lipid mixing by 6–7-fold. In the following, we derive a theoretical model that explains the experimental observation.

### A Theoretical Model of Stalk Energy and Tension Relation

Lipid mixing must follow the merger of the proximal monolayers of the fusing membranes and the formation of a hemifusion stalk (“stalk” for brevity), which is the initial lipidic connection between the membranes. Previous studies found stalk formation to be the major barrier to lipid mixing.^11,31^ Therefore, we speculated that the prolonged lipid mixing time delay is due to the increased stalk energy. To test this hypothesis, we calculated the dependence of the stalk energy on tension using continuum elastic theory.

We distinguish between two contributions to the stalk energy: *F*_0_ represents all terms independent of the tension increase, such as the elastic energy associated with lipid monolayer deformations, dehydration energy, and the contribution of residual tension originating from membrane thermal undulations, which is typically in the range of μN/m.^32^ The second contribution, *F*_T_, is due to the product of membrane area change and tension and is given by
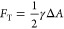
1Δ*A* is the sum of the area withdrawn from both monolayers to form the stalk (eq 14), Δ*A* = Δ*A*_distal_ + Δ*A*_proximal_, which we assume to share the tension equally, and γ is the membrane tension. The tension treated by our theory is in the 0.01–0.1 mN/m range, sufficiently above the thermally induced tension and below the critical rupture tension.

**Figure 4:**
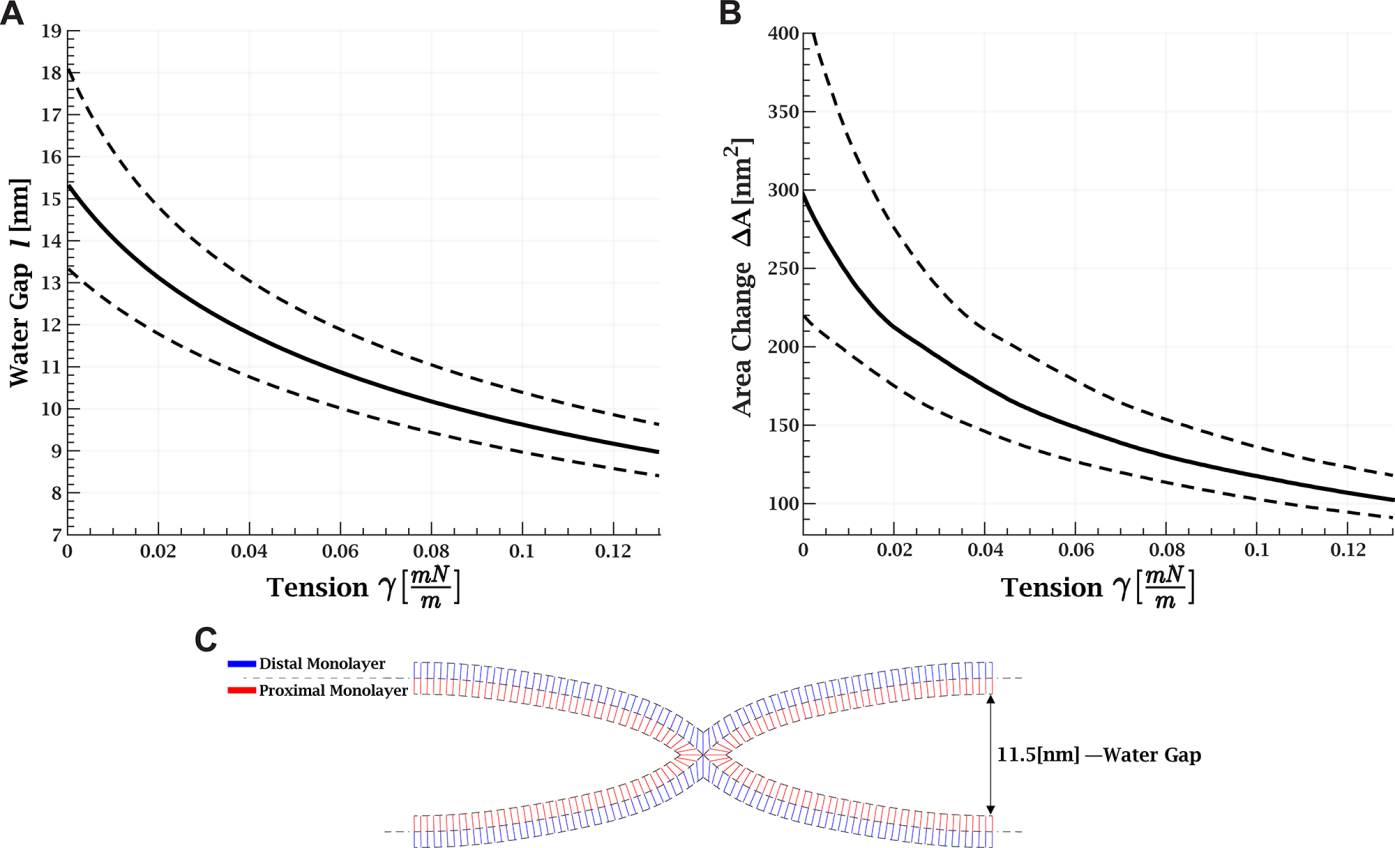
Stalk shape as a function of membrane tension. (A) Water gap between membranes as a function of membrane tension. (B) Monolayer area change, Δ*A*, as a function of tension. (A and B) Joining pressure is 1.57 Pa (full black line). The dashed line represents the error due to uncertainty in the joining pressure; the upper line corresponds to minimum pressure of 0.95 Pa, and the lower line to maximum pressure of 2.38 Pa. (C) Example of simulation result of stalk shape with a 11.5 nm water gap between the membranes. Parameters: monolayer bending rigidity of 17.5 *k*_B_*T*, monolayer saddle-splay modulus of −8.75 *k*_B_*T*, tilt rigidity of 40 mN/m, monolayer width of 1.5 nm, and spontaneous monolayer curvature of −0.18 nm^–1^.

**Figure 5:**
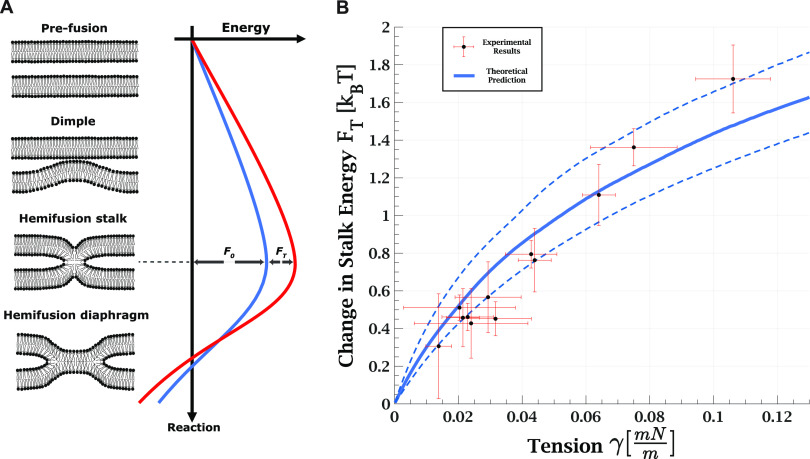
(A) Schematic illustration of the transition from pre-fusion to hemifusion state. The pre-fusion configuration is considered as two flat membranes whose distance is set by the balance between external forces pushing them together and repulsive undulation interaction. The second metastable configuration is a hemifusion state such as hemifusion diaphragm or elongated stalk, which are the minimal energy configurations at which the proximal monolayers are fused, but the distal monolayers are still separated. The hemifusion stalk represents the maximal energy along this pathway with the maximum amount of area pulled from the surrounding membranes and maximal elastic energy. *F*_0_ is the stalk energy independent of tension, and *F*_T_ is the tension-dependent term. (B) Change in energy barrier to lipid mixing: theoretical prediction versus experimental results. The scattered black dots (9 vesicles, 21 measurements) are the , with  being the ratio between lipid mixing time delay with tension γ to the lowest measured tension for each GUV. The error in the tension corresponds to the initial tension deviation from zero for the lowest tension measurement of the specific GUV. The continuous solid line is the theoretically predicted increase in the stalk formation energy due to tension. The dashed lines represent the validity limits of the theoretical prediction due to uncertainty in the external pressure. Bilayer bending rigidity is taken as 35 *k*_B_*T* for the theoretical prediction.

The contribution of tension to the stalk energy is governed by Δ*A*, which depends on the water gap between the membranes, *l*. We control the joining force of the membranes using optical tweezers and thus, indirectly, the distance. The distance before stalk formation is controlled by the balance between this force and the repulsive intermembrane interaction, which is dominated by thermal membrane undulations. The joining pressure is evaluated as 0.95–2.38 Pa with a mean value of 1.57 Pa from the contact area between the bead and GUV, 7.2 ± 1.0 μm^2^, and the pushing force, 11.3 ± 3.5 pN (see SI Appendix B, Figure S4). The full black lines in Figures 4A and B and Figure 5B correspond to the mean joining pressure value and the dashed lines for the maximal and minimal pressures, respectively.

The repulsive pressure originates mostly from membrane undulations and is derived in SI Appendix A. The tension decreases the undulations, and as a result, this repulsive pressure is reduced, a process known as tension-induced adhesion.^33^ The dependence is given by (SI Appendix A, eq 10)
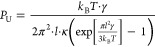
2The membrane bending rigidity, κ, is taken as 35 *k*_B_*T* for the cholesterol-rich (30%) lipid composition used here.^34−36^ As a result, the water gap equilibrium distance between the membranes is 15 nm without tension and is reduced by 6 nm at 0.12 mN/m (Figure 4A). At these ranges van der Waals, electrostatic, and hydration forces are negligible.

Next, we simulated the shape of the stalk based on the water gap distance using the continuum elastic approach.^11,31,37^ An example of the simulation results is presented in Figure 4C. We derived the total monolayer area change, Δ*A*, as a function of *l* (SI Appendix B, Figure S5, black) and the change in proximal and distal areas (Figure S5, blue and red, respectively). Finally, we calculated Δ*A* as a function of tension (Figure 4B) by considering the reduction in the water gap as the tension increases. We found that Δ*A* is reduced from 298 nm^2^ at a vanishing tension to 107 nm^2^ at 0.12 mN/m.

The contribution of tension to the stalk energy is found by inserting Δ*A* into eq 1. We find that at the maximal measured tension (0.12 mN/m) *F*_T_ is 1.6 *k*_B_*T*, an order of magnitude smaller than the tension-independent stalk energy, *F*_0_, previously found to be 20–60 *k*_B_*T*.^11,31,38,39^ However, the change in the lipid mixing time delay due to tension depends solely on the change in the energy barrier and not on its absolute magnitude, as explained in the following section.

### Dependence of Lipid Mixing Time Delay on Tension

We model lipid mixing as the transition from separated membranes to a metastable hemifusion state at which the proximal monolayers merge and lipids can exchange. The stalk is a necessary step with the highest energy along the pathway; its formation is the rate-determining step in the process. Following stalk formation, the excess energy is relaxed by either expansion to the hemifusion diaphragm^40^ or other intermediates, such as an elongated stalk.^41−44^ This process is depicted in Figure 5 A. The lipid mixing time delay is proportional to the first passage time over the energy barrier, which in our model is the stalk energy.

We consider the movement along the reaction coordinate as a diffusive process. The mean first passage time over the barrier between two metastable states is given by Kramer’s rate theory^45^ and is proportional to the exponent of the energy barrier,
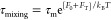
3*F*_T_ and *F*_0_ are the tension-dependent and -independent contributions to the stalk energy presented in the previous section; their sum is the energy barrier (Figure 5A). τ_m_ is an unknown microscopical time constant independent of the energy barrier, which our theory cannot predict without going into the molecular details.

We are interested in the contribution of tension to the lipid mixing time delay. Therefore, we eliminate τ_m_ and *F*_0_ by taking the ratio between the lipid mixing time delay in the presence of tension, τ_γ_, to the corresponding time without tension, τ_0_,
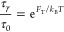
4To obtain a direct relationship between the time delay ratio, τ_γ_/τ_0_, to tension, we insert *F*_T_ (eq 1) and rearrange eq 4:
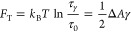
5In other words, the ratio between the time delays depends on the product of tension and the area change associated with stalk formation Δ*A*, calculated in detail in the previous section. We note that Δ*A* decreases with tension (Figure 4B) since the water gap distance between the membranes is reduced (Figure 4A). The resulting product, , monotonically increases with tension but with a decreasing slope (Figure 5B, blue line). This finding agrees with previous experiments showing that reduction in intermembrane distance accelerated lipid mixing between GUVs subjected to small tension.^27^

Finally, we compare this theoretical prediction to the experimental results by plotting the product of *k*_B_*T* and the experimentally measured logarithmic ratio of the lipid mixing time delay under tension to the time at vanishing tension, , as a function of the tension change for each GUV (Figure 5 B, black dots). We found excellent agreement between the experimental and theoretical results, even though no fitting was performed. Our results suggest that the increased stalk energy is the dominant factor contributing to the prolonged lipid mixing time delay.

### Biological Implications and Research Limitations

Fusion is a central physiological process occurring in all cellular membranes and over a wide range of tensions, typically much higher at the plasma membrane than in the internal organelles. The dynamics of membrane fusion are strongly affected by tension. While the transition from hemifusion to full fusion was previously found to be accelerated by tension,^25,26^ the tension effect on lipid mixing was hitherto less understood. We found that tension inhibits lipid mixing because it increases the stalk formation energy, which is a necessary step in the process.

Tension has a 2-fold effect on the stalk formation energy: on the one hand, tension increases the energy cost of withdrawing membrane area from the surrounding membranes. On the other hand, it lowers the undulations of the approaching membranes and, thus, their equilibrium distance, reducing the needed area for stalk formation (as seen in Figure 4B). Although the two effects work against each other, the first one dominates, resulting in an increase in the energy barrier, as seen in Figure 5 B, and hence a prolonged time to lipid mixing.

The range of tensions applied in our experiments is 0.005–0.12 mN/m (Figure 3B). In physiological membranes, tension ranges from 0.005 to 0.015 mN/m in the inner organelles, such as the endoplasmic reticulum and Golgi,^17^ to 0.5 mN/m in the plasma membranes of migrating cells.^19,20^ Based on our simplistic model and assuming a fixed separation distance of 10 nm (Δ*A* = 126 nm^2^, Figure S5), the tension contribution to the energy barrier is estimated as 0.08–0.23 *k*_B_*T* in inner organelles and up to 23 *k*_B_*T* in the plasma membrane. Therefore, the fusion machinery at the plasma membrane must exert stronger forces to achieve hemifusion at a time comparable to that of fusion in the internal organelles. Furthermore, it was recently discovered that SNARE proteins bring membranes to tight proximity as part of their fusion mechanism, which might be a possible pathway to overcome the high stalk energy induced by tension.^46^

However, we emphasize that our estimations should be considered semiquantitative since the results strongly depend on the intermembrane distance, which varies depending on the biological context and the fusion machinery. Moreover, our experimental setting allows us to set the tension in the GUV membrane but not in the bead, which acts as a tight membrane support. The presence of the support significantly increases the tension-independent contribution to stalk energy, *F*_0_, since the stalk formation involves local membrane detachment from the support.^47^ However, the tension-dependent term, *F*_T_, is not significantly affected; while a negligible amount of membrane is withdrawn from the supported membrane, almost double the amount is drawn from the free membrane, resulting in a similar Δ*A*. To estimate the correction, we assume that the stalk shape at the GUV side is unaffected by the bead support. In that case, the area correction to the tension-dependent term can be approximated by
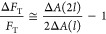
6At 0.05 mN/m, the middle of our experimental tension range, the maximal correction to the stalk energy due to the support is 50%, corresponding to ∼0.5 *k*_B_*T*. To compare, the *F*_T_ error due to pressure uncertainty and the variance in membrane bending rigidity, an important factor in our model, is of similar magnitude. Therefore, this correction is within the limits of our theoretical predictions.

We do not observe full fusion in our experimental system, probably because of the interaction of the membrane with the bead (Appendix B, Figure S6B). Previous experimental studies revealed asymmetry between the leaflets of a supported lipid bilayer, leading to a significantly lower lipid diffusion rate in the inner monolayer than in the outer monolayer.^48,49^ These results indicate a strong coupling between the support and the inner monolayer, which might increase the energy barrier of later fusion stages involving distal monolayer remodeling. Therefore, it is likely that the adhesion to the bead prevents full fusion.

The tension-dependent contribution to the stalk energy depends on the water gap between the fusing membranes with a larger gap resulting in a stronger influence of tension on the lipid mixing time delay. The lipid mixing time delay of membranes in tight docking is not expected to be significantly influenced by tension since the area change needed for stalk formation, Δ*A*, is small. In agreement with this prediction, previous computational works found that lipid mixing is not inhibited by tension in membranes that are strongly adhered prior to fusion.^22−24^ In contrast, our theoretical analysis predicts a membrane gap of 9–15 nm in our setting. At this distance, the interaction is dominated by the repulsive force of membrane undulations. In agreement with this prediction, X-ray scattering measurements of multilamellar membranes with a similar lipid composition and Ca^2+^ concentration found an intermembrane distance of ∼14 nm,^50^ indicating that the membranes do not adhere. Therefore, tension is an important factor in the stalk energy in our setting.

## Conclusions

Membrane tension energetically favors fusion but inhibits lipid mixing by increasing the stalk energy. These findings and the good agreement between our experimental and theoretical analysis results corroborate the hypothesis that stalk formation is the major barrier to lipid mixing when the pre-fusion membranes are not tightly docked. Hence, the fusion process might be inhibited or blocked by the high stalk energy in physiological membranes with high tension, such as plasma membranes. We also believe that our newly developed experimental setup can be used to advance the understanding of the mechanisms involved in specific situations of membrane fusion of high biological and medical relevance.

## Methods

### GUV Preparation

Chloroform stock solutions of 1,2-dioleoyl-*sn*-glycero-3-phosphocholine (DOPC, Avanti Polar Lipids), 1,2-dioleoyl-*sn*-glycero-3-phospho-l-serine (DOPS, Avanti Polar Lipids), and cholesterol (Sigma) were mixed at a final lipid concentration of 0.25 mM and labeled with 0.1% Rhodamine-PE (RH-PE, Avanti Polar Lipids). GUVs were grown on ITO slides (Nanion Technologies) by gently spreading 30 μL of the lipid solution and evaporating the solvent by an argon stream, followed by desiccation under a mild vacuum for at least 2 h. GUVs were then grown by electroformation in 275 mL of 300 mM sucrose solution using a Vesicle Prep Pro instrument (Nanion Technologies). First, the electroformation voltage was increased stepwise to 3 V, 15 Hz) and applied at 55 °C for 2 h, followed by a slow decrease of voltage and frequency (see SI Appendix B, Figure S1).

### Bead Coating

DOPC, DOPS, and cholesterol were mixed at a final lipid concentration of 0.25 mM in chloroform and labeled with 0.1% Oregon Green 488 1,2-dihexadecanoyl-*sn*-glycero-3-phosphoethanolamine (Oregon Green 488 DHPE; Invitrogen). The solvent was evaporated under an argon stream, followed by desiccation under a mild vacuum overnight. Next, the lipids were rehydrated with 1 mL of HEPES buffer (20 mM HEPES, 140 mM NaCl, 7.4 pH, 314 mOsmol). Liposomes were produced by extrusion through a 100 nm polycarbonate filter at 25 °C using a mini extruder (Avanti Polar Lipids). Polystyrene nonporous particles (beads; Spherotech Inc.) of 3.15 μm diameter were suspended in Milli-Q water and washed through 3 cycles of vortexing followed by centrifugation for 5 min at 1000*g*. Washed beads were introduced into a 600 μL liposome dispersion and continuously mixed on a rotator overnight to form a continuous lipid bilayer of the beads.^51^ Next, beads were washed three times with a HEPES buffer to remove any free liposomes.

### Optical Tweezers (OT)

The experiments were performed using a C-trap confocal fluorescence optical tweezers setup (Lumicks) made of an inverted microscope based on a water-immersion objective (NA 1.2) with a condenser top lens. The optical trap is generated by a 10 W 1064 nm laser. The displacement of the optically trapped beads from the center was measured and converted into a force signal by back-focal-plane interferometry of the condenser lens with a position-sensitive detector. The samples were illuminated by a bright field 850 nm LED and imaged in transmission onto a metal-oxide semiconductor camera (CMOS).

### Confocal Fluorescence Microscopy

The C-trap includes three fiber-coupled excitation lasers with 488, 561, and 638 nm wavelengths. Scanning is performed using a fast tip/tilt piezo mirror. For confocal detection, the emitted fluorescence was descanned, separated from the excitation by a dichroic mirror, and filtered using emission filters (blue: 500–550 nm; green: 575–625 nm; red: 650–750 nm). Photons were counted by using a fiber-coupled single photon counting module. The multimode fibers serve as pinholes, providing background rejection.

### Experimental Chamber

PDMS walls were placed on a glass slide (0.13–0.17 mm; BAR-NAOR Ltd.) and mounted onto an automated XY-stage. GUVs and coated beads were added into the chamber containing glucose buffer (20 mM HEPES, 20 mM NaCl, 260 mM glucose, 7.4 pH, 314 mOsmol) and allowed to settle for 15 min before the experiment. The 488 and 561 nm lasers were used for confocal imaging to excite Oregon Green and RH-PE (respectively), with emission detected in three channels (blue, green, and red).

### Micropipette Aspiration Setup and Hemifusion Measurements

A micropipette aspiration setup, including a micromanipulator (Sensapex) holding a capillary of 5 μm diameter (Biological Industries) connected to a pump (EZ-25; Fluigent), was integrated into our optical tweezers instrument. By controlling the aspiration pressure, membrane tension on the GUV was modified according to^52^
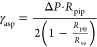
7γ_asp_ is the aspiration tension, Δ*P* is the micropipette suction pressure, *R*_ve_ is the vesicle radius, and *R*_pip_ is the micropipette radius. Before each experiment, the zero-suction pressure was found by aspirating a bead into the pipette and reducing the suction pressure until the bead stopped moving. For most experiments, multiple measurements under different tensions were performed on the same GUV (2–4 measurements).

For this assay, GUVs and membrane-coated beads were prepared with a composition of 50:30:20 (DOPC:cholesterol:DOPS). To initiate the hemifusion, Ca^2+^ was added with a concentration of 8.3 mM (Ca^2+^ concentration optimization shown in SI Appendix B, Figure S3). Then, the GUV and bead were brought in contact with an ∼11 pN force (see SI Appendix B, Figure S4B) while simultaneously monitoring the fluorescence intensity on the bead. This force was chosen since it allowed lipid mixing while maintaining the bead in the trap and was used in all measurements. We maintain a constant pushing force in our experiments since the effect of intermembrane distance on stalk formation has been previously addressed experimentally^53^ and theoretically.^31^

To ensure sealing between the GUV and the micropipette, the GUV was aspirated under a high tension of 0.2 mN/m before each measurement. Then measurements were performed at the desired suction pressure/tension values.

We note that other methods are available for modifying tension, such as osmolarity and the GUV radius change. A very recent development is the optical control of the membrane area using photoswitchable lipids.^54,55^ However, micropipette aspiration is a well-established method that allows direct control over tension.^52^ We validated our method by conducting bending rigidity measurements for pure DOPC membranes (SI Appendix B, Figure S2). In this experiment, a tether is pulled from an aspirated GUV with an optically trapped bead, followed by measuring the tether-pulling force under different membrane tensions. We found a bending rigidity of 18.0 ± 4.4 *k*_B_*T*, in agreement with the values previously reported in the literature.^35^

### Lipid Intrinsic Curvature Effect on Hemifusion

The effect of lipid composition on the lipid mixing time delay was tested by adding varying amounts of 14:0 LPC (positive intrinsic curvature; Avanti Polar Lipids) and cholesterol (negative intrinsic curvature). All the hemifusion measurements were performed under a mild tension of 0.04 ± 0.01 mN/m.

GUVs and membrane-coated beads were prepared for LPC measurements with a lipid composition of 50:30:20 (DOPC:cholesterol:DOPS). An LPC-supplemented buffer was prepared by rehydrating an LPC film (previously desiccated under a mild vacuum for 2 h) with a glucose buffer and further sonication for 1 min. The vesicles and beads were added to the experimental chamber containing an LPC-supplemented buffer (0, 5, and 10 μM) and allowed to settle for 15 min before the measurement. LPC concentration was below the CMC; therefore it easily inserted into the proximal monolayer.^56^ GUVs and membrane-coated beads were prepared for cholesterol measurements with a lipid composition containing different DOPC:cholesterol:DOPS ratios (80:0:20, 50:30:20, and 40:40:20).

### Experimental Data Analysis

Data analysis was carried out using Bluelake, a commercial software by Lumicks. The software stores experimental data acquired during the experiments in HDF5 file format, which can be processed by using Lumicks’ Pylake python package. Images of the confocal scans were reconstituted from the photon count per pixel data. All data analysis was performed with custom-written Python scripts.

### Calculation of Hemifusion Stalk Shape and Area Change

We use continuum elastic theory to calculate the stalk’s shape, as used previously, to address all intermediates in the canonical fusion pathway.^39^ In this approach, the stalk is the minimal energy shape at which the proximal monolayers are merged, while the distal monolayers are still separated.

To calculate the stalk shape, we fix the angle between the membrane midplanes to 90° at the center of the stalk to prevent voids between the hydrocarbon tails.^11^ As a result, the lipids are strongly sheared and splayed near the stalk. These deformations decay at a distance of a few nanometers, beyond which the membranes are flat and parallel. Furthermore, we explicitly prevent stalk expansion or elongation since we are interested in the highest energy intermediate determining the lipid mixing rate. The hemifusion diaphragm and elongated stalk have lower energy than the stalk^40,57^ and form downstream and, therefore, do not determine the lipid mixing time delay.

We consider two contributions to the stalk energy: elastic tilt-splay deformations of the lipid tails and the work related to pulling lipids from the surrounding membrane reservoir under tension. Lipid tilt is defined as , with *N⃗* normal to the lipid monolayer dividing plane and *n⃗* a vector pointing from the base to the head of the lipids. The lipid-splay is derived from the lipid-splay tensor, *b̃*_α_^β^ = ∇_α_*n*^β^, with the total lipid splay being its trance *J̃* = *b̃*_α_^α^ and lipid saddle splay its determinant *K̃* = det *b̃*_α_^β^. Without tilt, the total and saddle splays are the total and Gaussian curvature, respectively. The elastic energy per unit area of the deformed monolayer with respect to a flat tilt-less configuration is given by^58,59^
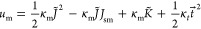
8κ_m_ and κ̅_m_ are the monolayer bending and saddle-splay moduli, taken as 17.5 *k*_B_*T*(35) and −8.75 *k*_B_*T*,^60,61^ respectively. The tilt modulus, κ_t_, is taken to be 40 mN/m.^62−65^ The spontaneous monolayer curvature, *J*_sm_, represents the preferred width of the lipid in the monolayer. It is given by the averaged sum over the lipid’s intrinsic curvatures:^66−68^
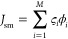
9with *M* the total number of lipid components, ζ_*i*_ the intrinsic curvature, and ϕ_*i*_ the mole fraction. The intrinsic curvature of cholesterol is −0.5 nm^–1^, DOPC is −0.09 nm^–1^,^69^ and DOPS is 0.07 nm^–1^.^70^ Based on these and the lipid mole fraction used, we set *J*_sm_ = 0.18 nm^–1^ in our simulations. The overall elastic energy is given by integrating the monolayer energy density from eq 8 over the areas of the two monolayers,
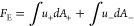
10*u*_±_ and *dA*_±_ are energy densities and monolayer dividing-plane area elements of the upper and lower monolayers, respectively.

The second contribution comes from the mechanical work of pulling lipids from the surrounding membranes to form the stalk against membrane tension. We consider the surrounding membranes to be much larger than the stalk and allow the lipids to freely exchange between the stalk in its vicinity and the surrounding membranes, which act as a reservoir. Such exchange is accompanied by thermodynamic work and, consequently, is related to the system’s free energy changes. This thermodynamic work per monolayer area is the monolayer tension and is defined as
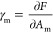
11The free energy derivative with respect to the monolayer area, *A*_m_, is taken while keeping all the other geometrical properties constant, including the area per lipid. As discussed in the introduction, the values of tension in cells vary within a broad range, but in general, they are orders of magnitude lower than the lipid area stretching modulus, which is in the range of 100–200 mN/m.^71^ Therefore, we simplify our analysis by taking the in-plane area per lipid as a constant and accounting only for the membrane area changes resulting from lipid exchange with the reservoir. The energy related to tension in the distal and proximal monolayers is given by
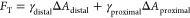
12Δ*A* is the monolayer area change with respect to the pre-fusion state. We assume in our computation that the tension is equal in both monolayers:
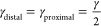
13γ being the membrane tension. Equation 12 then reads
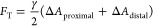
14The stalk energy is the minimum of the elastic (eq 10) and tension-related (eq 12) energies sum:
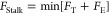
15We minimize eq 15 using a self-written software package (https://github.com/GonenGolani/Fusion_Solver). More details can be found in a previous publication^39^ and the code itself.

## Supporting Information Available

Supplementary Movie A: Membrane fusion at 20 mM Ca^2+^ for lipid composition as in Figure S3 (MP4)
Movie B: Hemifusion at different tensions for lipid composition as in Figure 3, at 8.3 mM Ca^2+^ (MP4)
SI Appendix A: Effect of tension on interaction forces between a fluctuating membrane and a rigid wall (PDF)
SI Appendix B: Supplementary figures (PDF)
