# Supplementary material for: Membrane Tension Inhibits Lipid Mixing by Increasing the Hemifusion Stalk Energy
Source: ACS Nano. 2023 Sep 5;17(19):18942–51. doi: 10.1021/acsnano.3c04293 (PMC7615193; doi:10.1021/acsnano.3c04293)
Supplement: Supplementary file 3 — nn3c04293_si_003.pdf [file nn3c04293_si_003.pdf]

Membrane Tension Inhibits Lipid Mixing by Increasing the Hemifusion Stalk Energy

SI Appendix B - Supplementary Figures

Petr Shendrik[#1,3], Gonen Golani[#2], Raviv Dharan[1,3], Ulrich S. Schwarz[2] and Raya Sorkin[1,3]*

[1] School of Chemistry, Raymond & Beverly Sackler Faculty of Exact Sciences, Tel Aviv University, Tel Aviv, Israel.

[2] Institute for Theoretical Physics and BioQuant Center for Quantitative Biology, Heidelberg University, Heidelberg, Germany.

[3] Center of Physics and Chemistry of Living Systems, Tel Aviv University, Tel Aviv, Israel.

*Corresponding author: rsorkin@tuaex.tau.ac.il

[#]These authors contributed equally

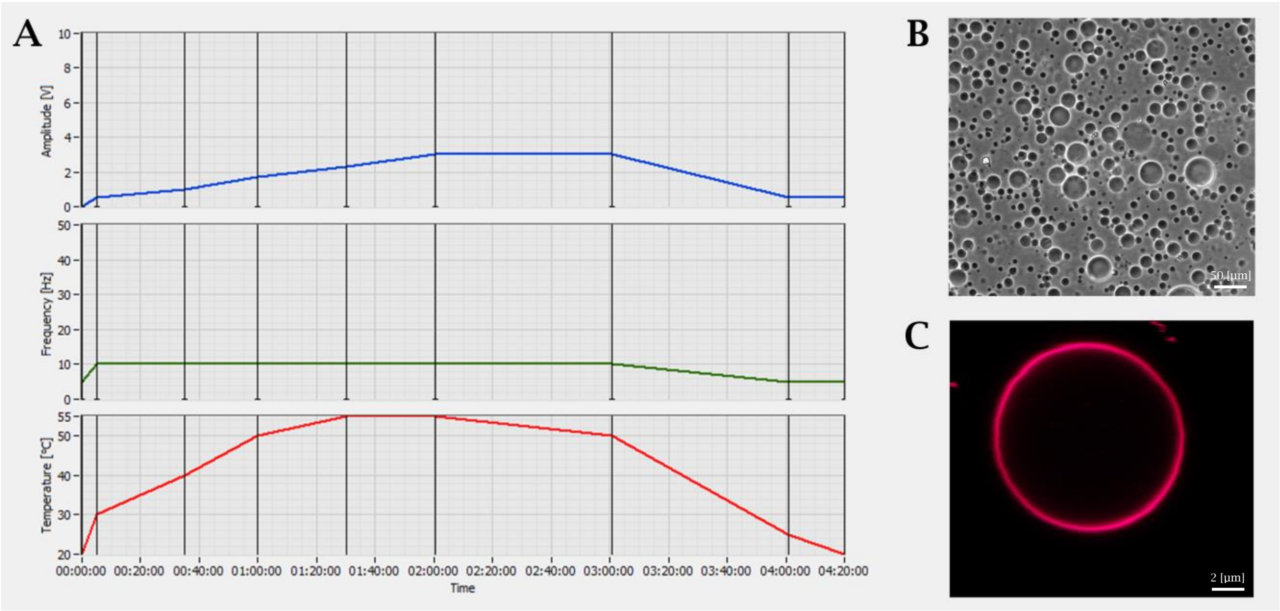

Figure S1 – GUV formation method. (A) Formation protocol for DOPS-rich GUVs. The voltage amplitude is increased stepwise to 3 V at a constant 10 Hz frequency, followed by a stepwise decrease. (B) Phase contrast image of formed GUVs (C) Confocal fluorescence image of a formed GUV.

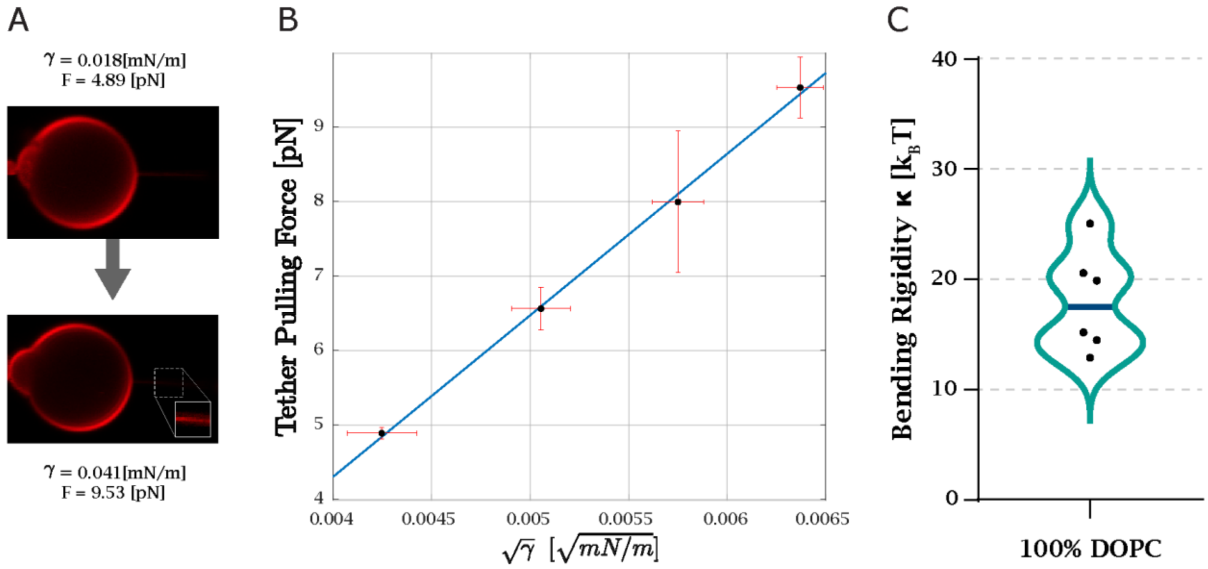

Figure S2 – DOPC GUV bending rigidity measurement using micropipette aspiration. (A) Confocal fluorescence images of the experimental setup. A tether is pulled with an optically trapped polystyrene bead from an aspirated GUV (99.9% DOPC, 0.1% RH-PE). The force exerted on the bead is monitored under different GUV tensions. (B) Tether pulling force as a function of the square root of tension, the blue solid line is a linear fit. The bending rigidity is evaluated from the slope[1]. (C) Bending rigidity of DOPC GUVs. The obtained value is 18.0±4.4 k_BT (23 measurements, 6 GUVs, from 3 independent experiments).

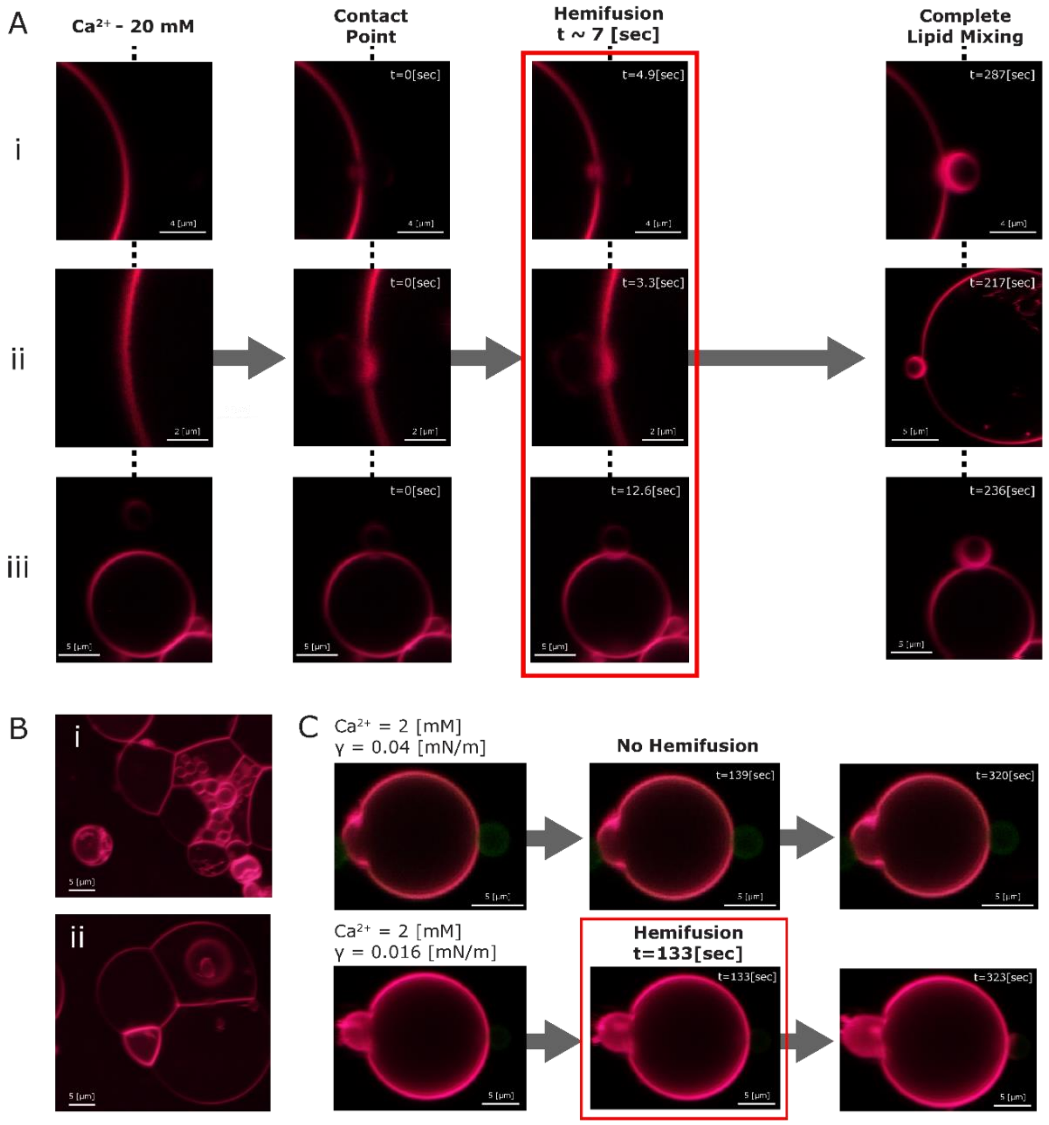

Figure S3 – Calcium concentration effect on lipid mixing of 80:20 (DOPC:DOPS) vesicles. (A) Lipid mixing measurements at 20 mM Ca^{2+} concentration. A membrane-coated bead is brought into contact with a free GUV, followed by fast lipid mixing initiation time of 7 sec (4 measurements). (B) (i-ii) Fluorescence images of GUV adhesion at high Ca^{2+} concentration (20 mM). (C) Lipid mixing measurements at 2 mM Ca^{2+} concentration. Most measurements resulted in no hemifusion (20/21 measurements, 4 independent experiments). The bottom row represents a successful hemifusion event at this concentration with 133 seconds lipid mixing time delay at 0.016 mN/m tension.

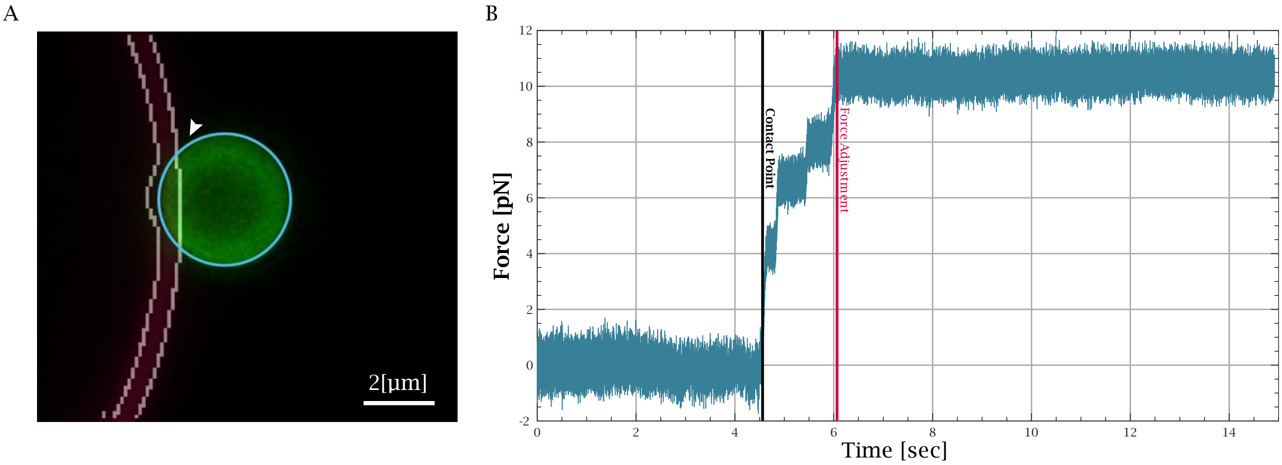

Figure S4 – Contact area and pushing force estimation in the experimental setup. (A) Confocal fluorescence image of a membrane-coated bead and a GUV after contact point. Both bead's (labeled with Oregon Green) and GUV's (labeled with RH-PE) edges are found from blue and red fluorescence channels, respectively, and the height of the bead part inserted into the GUV is evaluated. The resulting contact area is 7.2 ± 1 μm^2 (based on 24 Images). (B) Exerting force on the optically trapped bead in the x-axis during the experiment. A positive force value indicates a bead shift from the trap center to the right, hence a pushing force in the same direction. Before each measurement, the force is calibrated and normalized to 0 [pN] at the initial bead position. After the contact point, each bead position was adjusted to 11 pN pushing force.

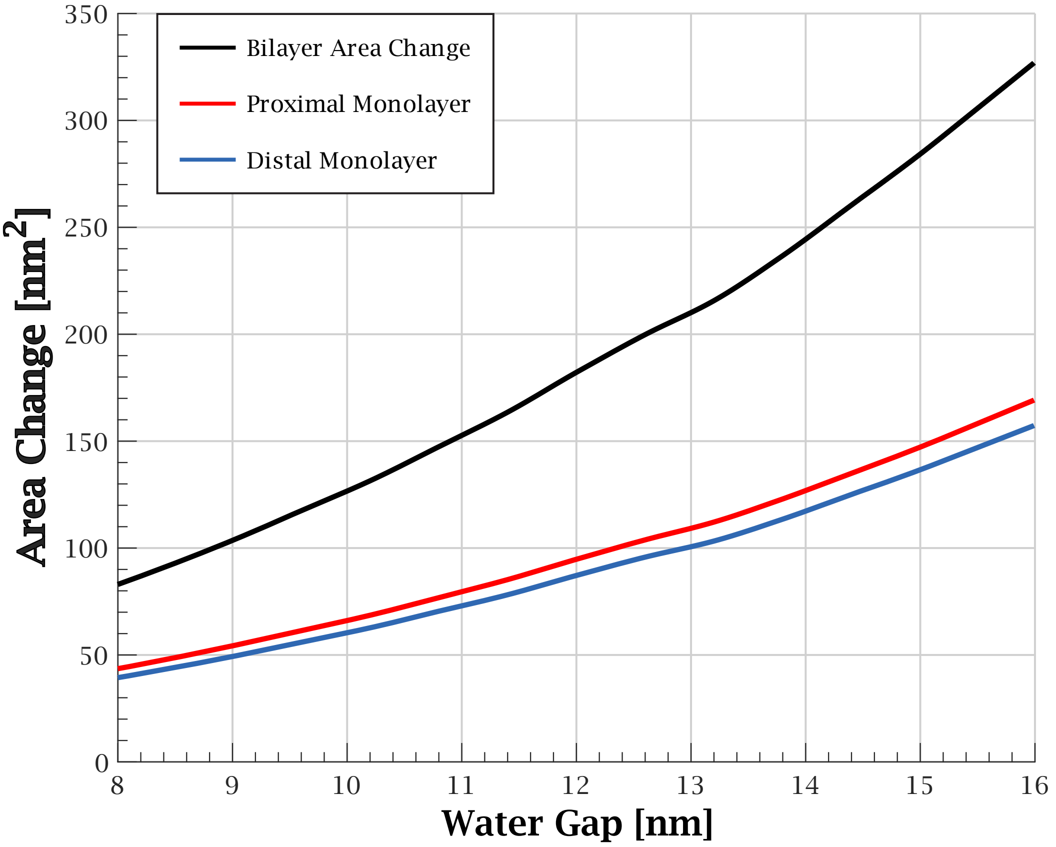

Figure S5 – Area withdrew from the membrane to form the stalk as a function of the water gap between the membranes. Parameters: Monolayer bending rigidity 17.5 k_BT, monolayer saddle-splay modulus -8.75 k_BT, tilt rigidity 40 mN/m, monolayer width 1.5 nm, and Spontaneous monolayer curvature -0.18 nm^{-1}.

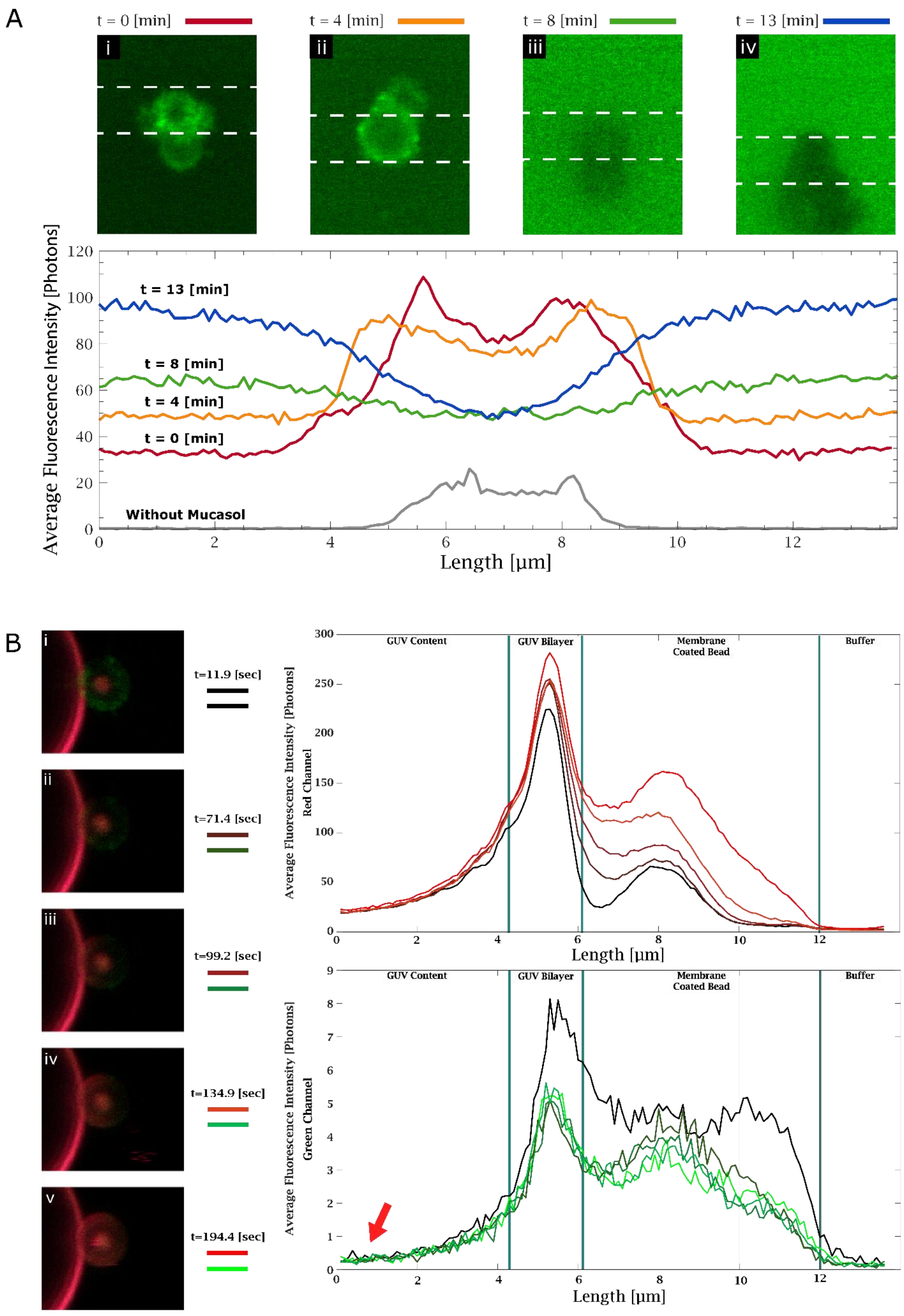

A
t = 0 [min]
t = 4 [min]
t = 8 [min]
t = 13 [min]
i
ii
iii
iv
Average Fluorescence Intensity [Photons]
t = 13 [min]
t = 8 [min]
t = 4 [min]
t = 0 [min]
Without Mucasol
Length [μm]
B
i
ii
iii
iv
v
t=11.9 [sec]
t=71.4 [sec]
t=99.2 [sec]
t=134.9 [sec]
t=194.4 [sec]
GUV Content
GUV Bilayer
Membrane Coated Bead
Buffer
Average Fluorescence Intensity [Photons]
Red Channel
Green Channel
Length [μm]

Figure S6 – Membrane-coated bead bilayer uniformity and fusion experiments. 6-Carboxyfluoresceine (CF6) at 50 mM concentration was encapsulated in the membrane-coated bead water layer by its addition to the formation buffer. (A) The fluorescence intensity on the beads was monitored by confocal fluorescence microscopy after an external addition of 0.5 %v/v detergent (Mucasol; Schuelke&Mayr). (i-iv) Fluorescence images of membrane-coated beads after mucasol addition; Initially, the fluorescence on the beads increased due to the dilution and dequenching of CF6, followed by slow increase in the fluorescence of the bulk. The bottom graph represents the averaged fluorescence intensity profile of the images within the range between dashed lines. The center of the graph is the profile on the bead, and the corners are the bulk. The grey line is the fluorescence profile of a bead before detergent addition. The intensity of the beads decreases with time while the intensity of the bulk increases. This result indicates that the CF6 was successfully encapsulated in the membrane-coated bead, demonstrating that the bilayer is continuous and uniform. (B) A CF6-encapsulated membrane-coated bead was brought into contact with an aspirated GUV to initiate the fusion process. The fluorescence intensity in the GUV interior was monitored during the lipid mixing to detect CF6 release and unquenching due to pore formation. (i-v) Fluorescence images of membrane-coated beads from the contact point to complete lipid mixing. The upper graph represents the average fluorescence intensity in the red channel (RH-PE lipid fluorescence). The intensity of the bead increases over time due to lipid mixing. The bottom graph represents the intensity in the green channel (CF6 fluorescence). In the beginning, the fluorescence on the bead decreases due to the bleaching of the quenched CF6, which has mild fluorescence. Over time, there is no change in the fluorescence in the GUV interior (red arrow on the left side of the graph), indicating no pore formation during the experiment. No fusion and pore formation was observed in 29 measurements from 6 independent experiments.

(1) Cuvelier, D.; Derényi, I.; Bassereau, P.; Nassoy, P. Coalescence of membrane tethers: experiments, theory, and applications. *Biophysical journal* **2005**, *88* (4), 2714-2726.
