# Supplementary material for: Membrane Tension Inhibits Lipid Mixing by Increasing the Hemifusion Stalk Energy
Source: ACS Nano. 2023 Sep 5;17(19):18942–51. doi: 10.1021/acsnano.3c04293 (PMC7615193; doi:10.1021/acsnano.3c04293)
Supplement: Supplementary file 4 — nn3c04293_si_004.pdf [file nn3c04293_si_004.pdf]

Membrane Tension Inhibits Lipid Mixing by Increasing the Hemifusion Stalk Energy

SI Appendix A – Effect of tension on interaction forces between a fluctuating membrane and a rigid wall

Petr Shendrik[#1,3], Gonen Golani[#2], Raviv Dharan[1,3], Ulrich S. Schwarz[2] and Raya Sorkin[1,3]*

[1] School of Chemistry, Raymond & Beverly Sackler Faculty of Exact Sciences, Tel Aviv University, Tel Aviv, Israel.

[2] Institute for Theoretical Physics and BioQuant Center for Quantitative Biology, Heidelberg University, Heidelberg, Germany.

[3] Center of Physics and Chemistry of Living Systems, Tel Aviv University, Tel Aviv, Israel.

*Corresponding author: rsorkin@tuaex.tau.ac.il

[#]These authors contributed equally

Membranes in close proximity repel each other due to the excluded volume effect of their mutual fluctuations. Tension diminishes these fluctuations and reduces the repulsive pressure. The following derivation follows the seminal work of Helfrich and Servuss[1, 2], who calculated the undulation magnitude, pressure, and interaction energy by considering only the bending rigidity of the membranes. Here we accounted for the contribution of tension. For the sake of brevity, we explicitly follow all major derivation steps, most of which can also be found in previous publications[1, 2].

We consider a flat membrane subjected to tension γ and bending rigidity κ. The Helfrich Hamiltonian [3] describes the energy in the Monge representation,

$$F = \int \left[\frac{1}{2}\kappa(\Delta u)^2 + \frac{1}{2}\gamma\left(\vec{\nabla}u\right)^2\right] dA, \tag{1}$$

with $u(\vec{r})$ the height of the membrane mid-plane at position $\vec{r} = x\hat{x} + y\hat{y}$, $\vec{\nabla}u$ is the height gradient and Δu is its Laplacian. The contribution of Gaussian curvature is a topological constant and is omitted, and we took the limit of a small gradient $\left|\vec{\nabla}u\right| \ll 1$. By using the equipartition theorem, the square of the magnitude of membrane fluctuations is given by,

$$\langle u_{\vec{q}}^2\rangle = \frac{k_B T}{A(\kappa q^4 + \gamma q^2)} \tag{2}$$

with $\vec{q} = \frac{2\pi}{\sqrt{A}}(m, n)$ being the wave number, and m and n are natural numbers. A is the membrane element area, which is much larger than the area of a single lipid, a. Under this assumption, the fluctuation magnitude in real space is given by

$$\langle u^2\rangle = \sum_{q_{min}}^{q_{max}}\langle u_{\vec{q}}^2\rangle = \frac{k_B T}{4\pi\gamma}\ln\left(1 + \frac{A\gamma}{\pi^2\kappa}\right) \tag{3}$$

Here we took only the contribution of long wavelengths and omitted contributions from short wavelengths in the order of a lipid headgroup radius. To find the pressure generated by the membrane fluctuations, we use a simple physical argument presented in previous works[2, 4, 5] – The membrane is considered to be between two rigid walls at a distance of $2l$, so the fluctuations are constricted to $h^2(\vec{r}) \leq l^2$. The membrane is segmented to area elements A_{eff}, each of which fluctuates independently and is considered a particle of a one-dimensional ideal gas. The average force such a particle exerts on the boundaries is $k_B T/2l$, and the pressure is given by

$$P = \frac{k_B T}{2 \cdot l \cdot Aeff}. \tag{4}$$

To find A_{eff}, we consider no correlations between modes and write the relation between the mean square amplitude and the wall boundaries as

$$\langle u^2\rangle = \alpha \cdot l^2. \tag{5}$$

α is a numerical pre-factor determining the reduction in mean square amplitude due to the presence of the walls. Inserting Eq. 3 to Eq. 5 and inverting we have,

$$A_{eff} = \frac{\pi^2\kappa}{\gamma}\left(\exp\left[\frac{4\pi\alpha l^2\gamma}{k_BT}\right] - 1\right). \quad (6)$$

The pressure is given by inserting Eq. 6 into Eq. 4:

$$P_U = \frac{k_BT\cdot\gamma}{2\pi^2\cdot l\cdot\kappa\left(\exp\left[\frac{4\pi\alpha l^2\gamma}{k_BT}\right]-1\right)} \quad (7)$$

In the small tension limit, $\frac{4\pi\alpha l^2\gamma}{k_BT} \ll 1$, we can approximate Eq. 7 to

$$P_U = \frac{(k_BT)^2}{8\pi^3\alpha\cdot\kappa\cdot l^3} - \frac{k_BT}{4\pi^2\cdot\kappa\cdot l}\gamma. \quad (8)$$

The first term in Eq. 8 corresponds to the tension-less pressure obtained By Helfrich and Servuss[2], and the second term is the first-order reduction in pressure due to tension. The pre-factor α determines, to a first-order approximation, the tension-independent contribution to the pressure. The lower limit to α can be obtained by restricting $\langle u^2\rangle$ to the interval between the walls and exciting only one mode. In this case, it can be shown that α equals 1/12. The upper limit is obtained by considering the membrane to be fixed at a single point. In such a case, α is 1/3. Helfrich and Servuss[2] estimated α as the geometric mean of the two limits, 1/6. However, it was argued that the interaction strength should be doubled if mode-mode correlations are considered [2]. Therefore, α has an additional ½ factor, resulting in α being equal to 1/12. The resulting tension-free repulsive interaction energy per unit area

$$\Delta G_{\gamma=0} = \int_{l'=\infty}^{l'=l} P_{\gamma=0}(l')dl' = \frac{3}{4\pi^3}\frac{(k_BT)^2}{\kappa\cdot l^2}. \quad (9)$$

Estimating the pre-factor can also be based on the undulation repulsive interaction energy between membranes at the multi-lamellar phase. Based on theoretical considerations, the pre-factor in Eq. 9 for membrane stacks was calculated as $3\pi^2/128\sim0.23$ [1] and by Monto-Carlo-based simulations as 0.11-0.074[6, 7]. Based on more sophisticated variational perturbation theory, the pre-factor was also estimated to be between 0.082-0.077[8, 9]. These numbers are larger than the pre-factor used here, $3/(4\pi^3)\sim0.024$. However, we consider the case of a membrane constricted by a single wall and not two walls, as discussed in these works. Therefore, amplitude reduction is significantly lower than in the case of a membrane stack, at least by a factor of 2. Therefore, we keep with the simple argumentation above and take α to be in the lower limit, 1/12. With that, Eq. 7 can be rewritten as

$$P_U = \frac{k_BT\cdot\gamma}{2\pi^2\cdot l\cdot\kappa\left(\exp\left[\frac{\pi}{3}\frac{l^2\gamma}{k_BT}\right]-1\right)}. \quad (10)$$

These estimates are, of course, rough, and the numerical results should be considered semi-quantitative.

(1) Helfrich, W. Steric interaction of fluid membranes in multilayer systems. *Zeitschrift für Naturforschung* **1978**, *33*, 3015-3315.
(2) Helfrich, W.; Servuss, R.-M. Undulations, steric interaction and cohesion of fluid membranes. *Il Nuovo Cimento D* **1984**, *3*, 137-151.
(3) Helfrich, W. Elastic properties of lipid bilayers: theory and possible experiments. *Zeitschrift für Naturforschung C* **1973**, *28* (11-12), 693-703.
(4) Lipowsky, R. Generic interactions of flexible membranes. *Handbook of biological physics* **1995**, *1*, 521-602.
(5) Israelachvili, J. N.; Wennerstroem, H. Entropic forces between amphiphilic surfaces in liquids. *The Journal of Physical Chemistry* **1992**, *96* (2), 520-531.
(6) Gompper, G.; Kroll, D. Steric interactions in multimembrane systems: a Monte Carlo study. *Europhysics Letters* **1989**, *9* (1), 59.
(7) Janke, W.; Kleinert, H. Fluctuation pressure of a stack of membranes. *Physical review letters* **1987**, *58* (2), 144.
(8) Kastening, B. Fluctuation pressure of a fluid membrane between walls through six loops. *Physical Review E* **2006**, *73* (1), 011101.
(9) Bachmann, M.; Kleinert, H.; Pelster, A. Fluctuation pressure of a stack of membranes. *Physical Review E* **2001**, *63* (5), 051709.
